# Silicate Rings in Woody Biomass Ash Melts Based on Molecular Dynamics Simulations

**DOI:** 10.1021/acsomega.5c12582

**Published:** 2026-03-08

**Authors:** Charlie Ma

**Affiliations:** Thermochemical Energy Conversion Laboratory (TEC-Lab), Department of Applied Physics and Electronics, 98833 Umeå University, Umeå SE-901 87, Västerbotten, Sweden

## Abstract

The medium-range ordering of the molten CaO–K_2_O–SiO_2_ system was investigated by studying the primitive silicate rings extracted from molecular dynamics simulations. Variations in the ring size distribution and the interconnectivity between the rings due to the different contents of the basic cations (Ca^2+^ and K^+^) were identified. With increasing basic cation content, i.e., depolymerization, the overall number of rings decreased, but increasingly larger rings (>10-membered) occurred while small-sized rings (4- to 7-membered) remained the most common. The share of 5-membered rings increased with depolymerization, particularly with K content. In terms of ring structure, compositions with more K cations also tended to produce rings with longer Si–O bonds and wider Si–O–Si angles; i.e., rings that were weaker and more stretched. A method was introduced to characterize the interconnectivity between rings, which indicated that the most common small-sized rings have a tendency to be interconnected to rings of specific larger sizes, depending on the composition. Visualization and counting of the basic cations inside of these rings showed that they were cation clusters. It suggests that these basic cation clusters tend to be located adjacent to Si-rich regions where small-sized rings interconnect with each other.

## Graphical abstract

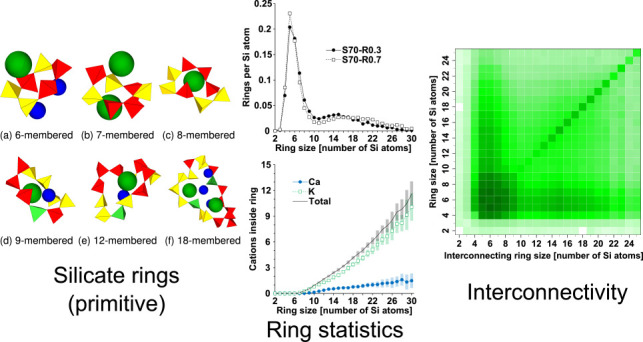

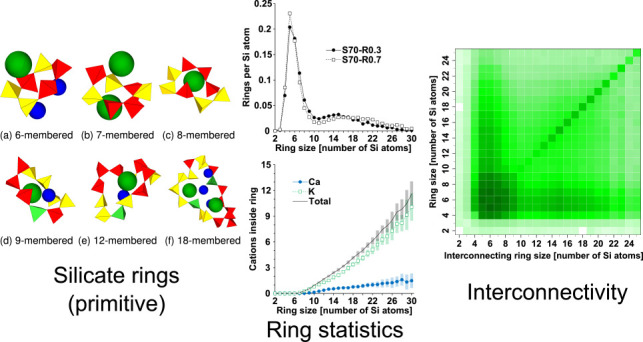

## 1 Introduction

Silicate-based molten ashes of solid fuels can often cause agglomeration, slagging, and blockage problems in thermochemical processes due to their viscous behavior.^1^ Like other types of silicates, molten silicates can be characterized at the molecular scale by different extents of structural ordering, in particular between Si and O atoms. The short-range ordering, as governed by the nearest-neighbor bonding between these atoms, forms the foundation of SiO_4_ silicate tetrahedron units. These silicate units can polymerize to form structures as part of networks that possess ordering. Long-range ordering of the networks—i.e., structural periodicity that is present in crystals—is not present in silicate melts and glasses, which are amorphous or topologically disordered.^2^ However, they do possess medium-range (also sometimes known as intermediate-range) structural ordering in the form of cross-linkages and discontinuities in the network, which can influence material properties, such as mechanical strength, electrical conductivity, and viscosity.^3–7^

The medium-range ordering underpins the modified random network model that depicts the underlying structure of multicomponent amorphous and liquid silicate systems as aperiodic networks of interconnected silicate tetrahedra interspersed by clusters of network-modifier cations.^8–11^ The modifier cation clusters create discontinuities in the silicate network and can consist of basic cations from common ash-forming elements, e.g., Ca, K, and Mg.^12^ A feature of the medium-range ordering is silicate rings (SiO_4_ tetrahedra connected in a circuitous path) that encircle such cation clusters. Statistics describing the sizes and quantities of these rings can thus characterize the extents of network cohesion, as well as the sizes of cation clusters.^13^ However, with the exception of small rings consisting of up to approximately six Si atoms,^14–16^ the experimental detection and quantification of larger rings is currently unattainable. On the other hand, computational methods, e.g., molecular dynamics (MD) simulations, enable them to be identified, analyzed, and classified in the most accessible manner, albeit numerically.^9,17^ Topological methods, such as persistent homology, have been applied to characterize the medium-range ordering in attempts to establish connections with experimental measurements.^18,19^

The focus of this study is the medium-range ordering of the CaO–K_2_O–SiO_2_ system, compositions of which are representative of ash slags from renewable woody biomass fuels.^12^ The objective was to elucidate the effect that the basic cations, Ca and K, have on the medium-range ordering of the silicate network. The network topology, in terms of the interconnectivity between the rings, was also studied in order to explore aspects of the spatial relationships between the basic cation clusters and the underlying silicate network.

## 2 Method

### 2.1 Computational Details

The BMP-harm interatomic potential function from the work of Bertani et al.^20^ was used in this study. It was developed from the Morse-based potential by Pedone et al.^21^ that has been widely used in MD simulations of multicomponent glasses and melts, including the conductivity of highly basic molten slags^22^ and the diffusion of mixed alkali/alkaline-earth silicate glasses.^23^ The potential includes a Buckingham repulsive term between network-forming atom pairs (i.e., Si–Si) and a nonbonded 3-body harmonic term for network-former bridging angles (i.e., Si–O–Si).^24^

The simulations were carried out using DL_POLY_4.10.^25^ The long-range Coulombic electrostatic forces were evaluated using the Ewald method with a cutoff at 12 Å, while the short-range force cutoff was at 7 Å. The atoms were simulated in timesteps of 1 femtosecond (fs), and slag densities from Lange^26^ were used to fix the initial volumes of the periodic cubic simulation domains. Random initial positions were assigned to the atoms before canonical (NVT) simulations at 5000 K were run for 10^5^ timesteps (100 ps) in order to relax and randomize the system. The temperature was then ramped down to the specified value at 5 K/ps and then to a pressure of 1 atm over 5 × 10^4^ timesteps using Nose–Hoover thermo- and barostat; i.e., the isothermal–isobaric (NPT) ensemble. Another 5 × 10^5^ timesteps (500 ps) were run before production, and data for analysis were dumped periodically over 3–250 ns, depending on the composition. The duration of each simulation was sufficiently long for the slowest diffusing atomic species (Si) to reach the diffusion regime.^27,28^ This was intended for the molecular structures to change sufficiently such that the averaged statistics extracted from the simulations would be representative of steady-state equilibrium, allowing the differences between the compositions to be clearly elucidated. The cubic periodic simulation domains were sufficiently large such that the rings found within a 3 × 3 × 3 duplicated domain supercell did not consist of duplicated counterpart atoms; i.e., maximum ring sizes were within the confines of each domain. The compositions of the CaO–K_2_O–SiO_2_ ternary slags are from the viscosity study by Chen and Zhao,^29^ which are representative of woody-type biomass ashes that are often characterized by high CaO and K_2_O contents with possible extensive inclusions of SiO_2_ from soil minerals.^12^ A pure SiO_2_ melt was included to serve as a reference of a fully polymerized system. Two binary CaO–SiO_2_ and K_2_O–SiO_2_ compositions were also included to aid comparison between the basic cations. The details of these compositions and other simulation inputs are listed in Table 1. The compositions S60C and S100 possess higher melting points, which meant that their simulations were carried out at 1500 and 1776 °C, respectively.

**Table 1:** Simulation Input Details^a^

	mol % (wt %)			*T* (°C)	simulation input atom quantities					simulated NPT box length (Å)
composition label	SiO_2_	K_2_O	CaO		O	Si	K	Ca	total	
S50-R0.3	49.8 (46.3)	17 (24.8)	33.2 (28.8)	1300	14,036	4666	3186	3111	24,999	73.4 ± 0.2
S53-R0.6	52.6 (45.5)	29.5 (40.0)	17.9 (14.5)	1300	12,442	4289	4810	1459	23,000	75.9 ± 0.3
S60K	60 (48.9)	40 (51.1)	0 (0)	1300	7467	2800	3734	0	14,001	65.9 ± 0.3
S60C	60 (61.7)	0 (0)	40 (38.2)	1500	9232	3462	0	2308	15,002	60.4 ± 0.2
S70-R0.3	70 (66.9)	10.5 (15.7)	19.5 (17.4)	1300	9090	3743	1122	1043	14,998	61.7 ± 0.3
S70-R0.7	70 (62.8)	21.1 (29.7)	8.9 (7.5)	1300	8760	3607	2174	459	15,000	63.8 ± 0.3
S78-R0.7	77.7 (71.8)	15.1 (21.9)	7.2 (6.2)	1300	7890	3450	1340	320	13,000	59.5 ± 0.3
S78-R0.4	78.2 (75.6)	7.6 (11.6)	14.2 (12.8)	1300	8106	3557	692	646	13,001	58.1 ± 0.3
S100	100 (100)	0 (0)	0 (0)	1776	1666	833	0	0	2499	32.6 ± 0.6

a The composition labels indicate S = SiO_2_ and the mol % content followed by K = K_2_O or C = CaO for the binary compositions or R=K2O(CaO+K2O) ratio for the ternary compositions.

R programming scripts were used to manage simulation procedures and to search for primitive rings based on positional data that were dumped periodically from the simulations. These results were compiled and averaged from analyzing at least 10^5^ Si atoms derived from entire configurations at different timesteps throughout the production stage of the simulations.

### 2.2 Ring Definition and Counting Procedure

In networks with significant connectivity, rings consisting of arbitrarily large numbers of Si atoms can be traced and identified. This arbitrariness implies that not all rings are meaningful toward characterizing the structure of the network nor helpful toward explaining the physical properties of the material. Yuan and Cormack^13^ discussed the merits of different ring definitions that are commonly used and recommended primitive rings as the most suitable to detect network-modifier clusters. Primitive rings are those that cannot be decomposed into two smaller rings; or equivalently, they contain no pathways between any of its members that consist of less members than that along the ring pathway in question, i.e., no shortcuts.^30^

In this study, primitive rings across specified size ranges were identified in each simulation. Every pair combination of the links emanating from each and every Si atom was looped through to identify these rings. This meant that each and every *n*-membered primitive ring was counted *n* times, i.e., rings were overcounted with increasing ring size. The total quantities of each ring size were therefore divided by their respective size to allow for fair comparison between the ring sizes. Note that this adjustment is not undertaken in some previous works (see Supporting Information Figure S1).^13,9^ The divisibility of the total numbers by each respective ring size also served as a check that the rings were counted correctly. Visualizations of the rings and cations were facilitated by the R library r gl.

## 3 Results and Discussion

### 3.1 Variation in Ring Distribution during Simulation

The ring size distributions from each simulation varied between configurations at different timesteps, as demonstrated by the composition S70-R0.3 in Figure 1. The variations do not vary systematically with the ring size, but some larger ring sizes can be notably more abundant for certain timesteps. This is because the probability of the presence of multiple pathways increases with increasing ring size. An example is given in Figure 2 for the gray SiO_4_ unit atom that possesses a branch pair from which eight 15-membered primitive rings can be traced. Multiple pathways containing the same number of SiO_4_ units that traverse between certain members (marked with ‘∗’) enable this to occur. Note that none of them constitute shortcuts; every 15-membered ring, including the smaller rings, are all primitive.

**Figure 1:**
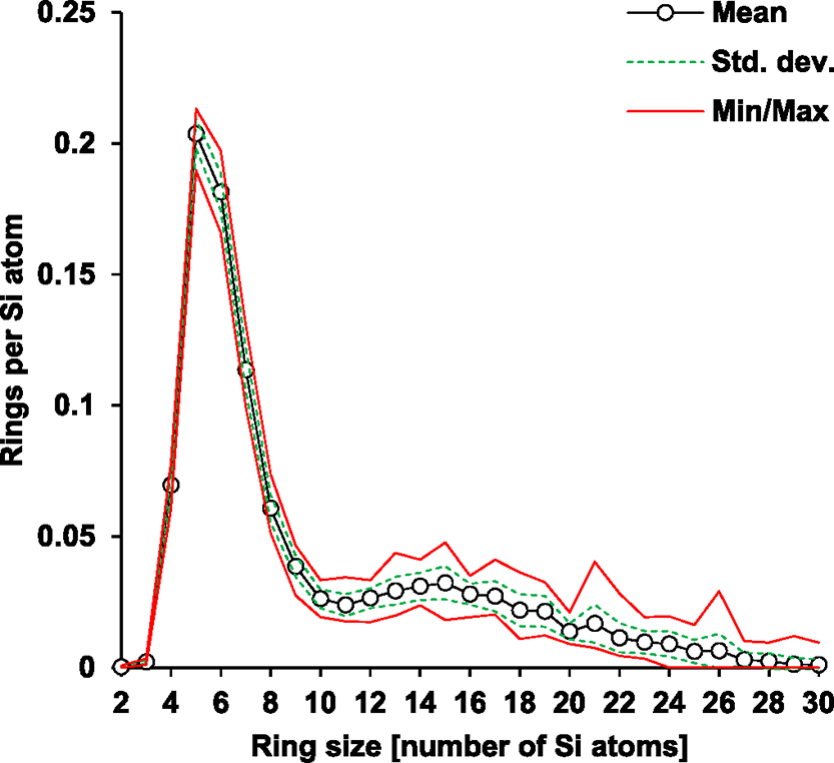
Distribution of primitive ring sizes for S70-R0.3 at 1300 °C. The statistics are based on variations of the ensemble at different timesteps throughout the simulation.

**Figure 2:**
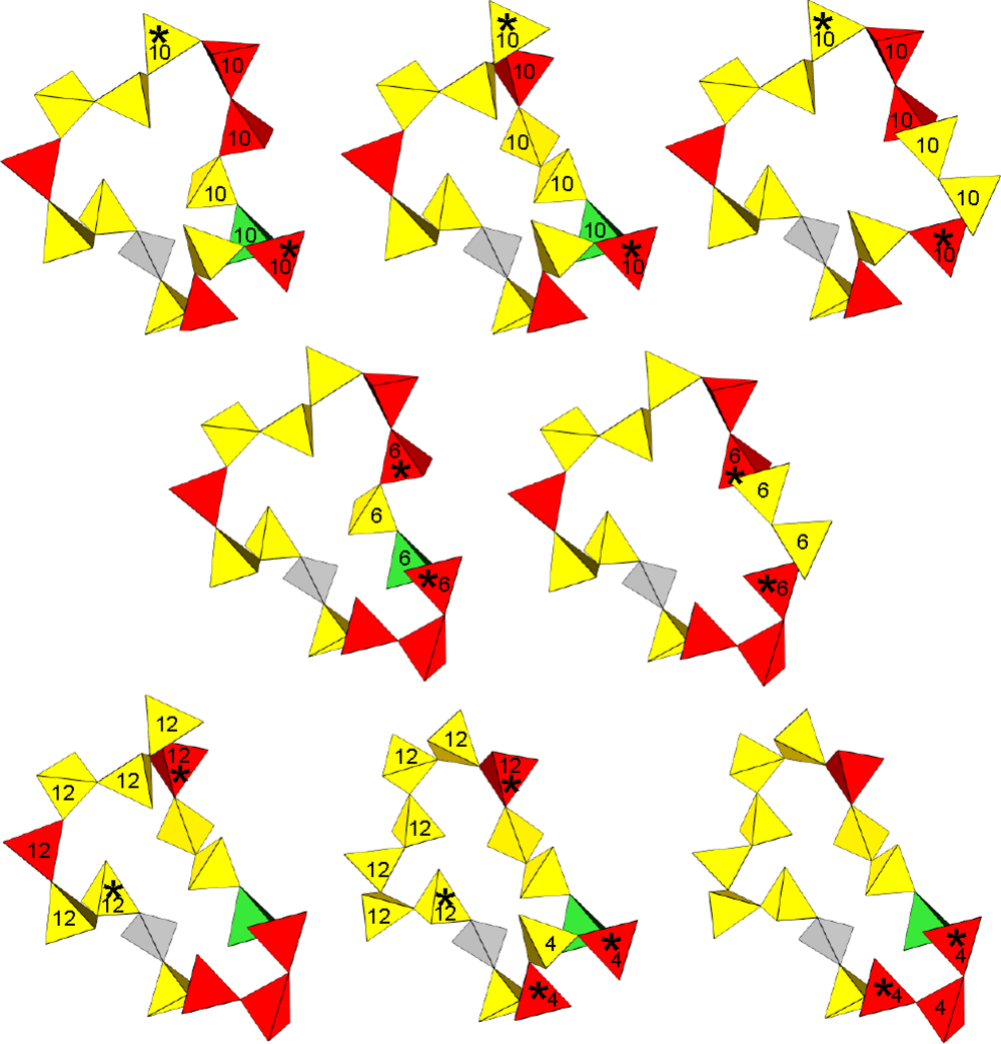
Multiple 15-membered primitive rings stemming from a single branch pair belonging to the gray SiO_4_ tetrahedron unit. Each tetrahedron represents a SiO_4_ unit, with its vertices at the centers of the O atoms that are coordinated to each Si within the tetrahedron. Their color indicates the *Q*_*n*_ value of the Si atom: *Q*_4_ (red), *Q*_3_ (yellow), and *Q*_2_ (green). The numbers indicate the sizes of smaller rings that the tetrahedra are members of that form alternate pathways between the tetrahedra marked with ‘∗’. Note that the labels for an additional 6-membered ring were omitted for the sake of clarity.

By averaging over a large number of Si atoms (>10^5^) from numerous timesteps during which all atoms have sufficient opportunity to diffuse and affect changes in the network structure, the inherent differences between the compositions were distinguished. Hence, only the averaged distributions are shown hereafter.

### 3.2 Effect of Composition on Ring Distribution

Figure 3a compares the distribution of relatively small primitive rings (≤16-membered) in a number of compositions with varying SiO_2_ content. The pure liquid SiO_2_ has a primitive ring size distribution that spans between 2 and 12 members, with the most abundant being 6- and 7-membered rings. This is in contrast to crystalline quartz SiO_2_, where each Si atom is associated with 6 × 6-membered and 40 × 8-membered primitive rings.^31^ For the compositions that contain the basic cations, the number of relatively small rings (≤10-membered) decreases with decreasing SiO_2_ content. It is clear that depolymerization decimates the silicate network cohesion, as characterized by the abundance of small rings, while facilitating the occurrence of larger rings (>10-membered).

**Figure 3:**
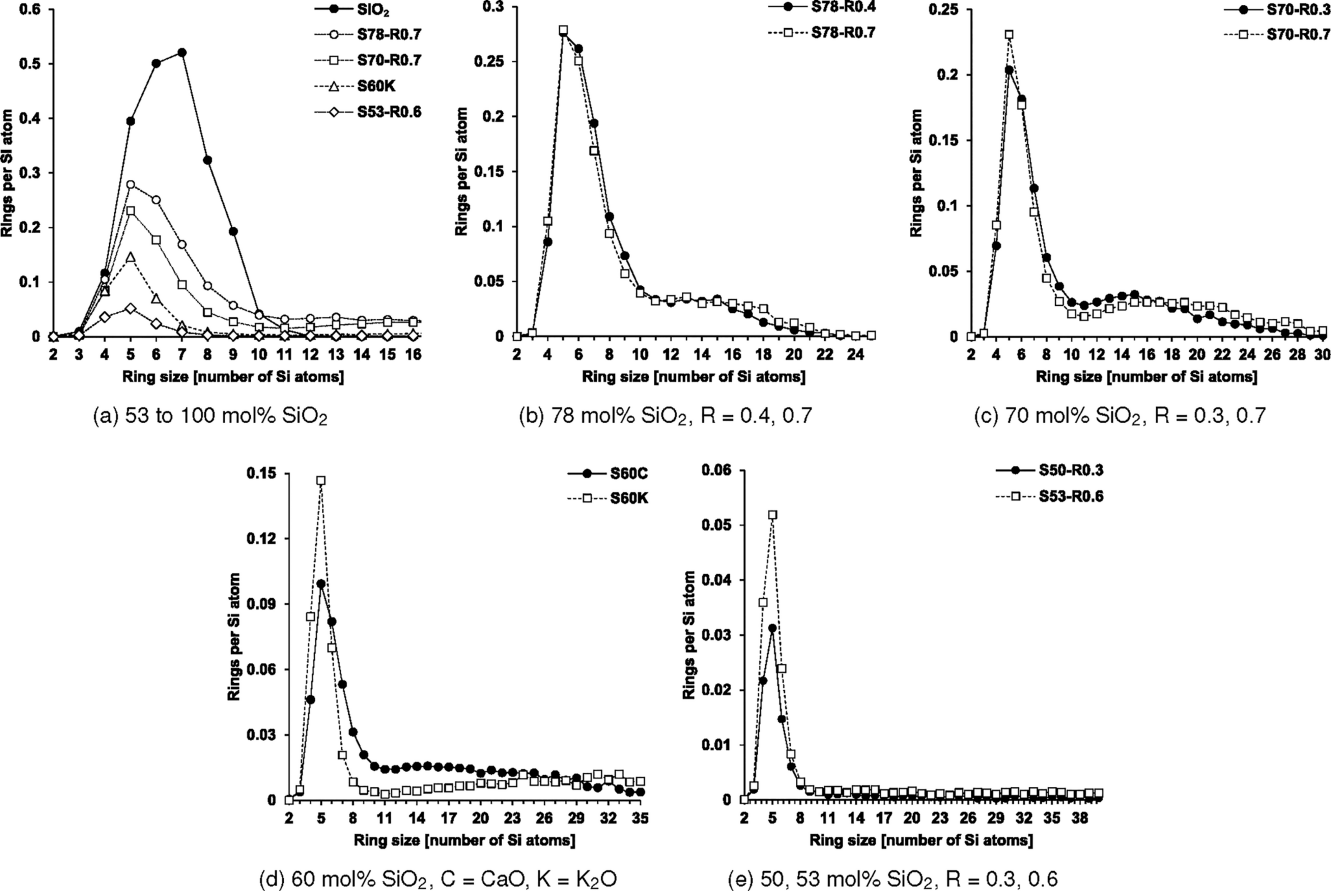
Comparison of ring size distribution with different SiO_2_ contents and basic cation ratios R=K2O(CaO+K2O) .

The more extensive ring size distributions of these compositions are compared based on their SiO_2_ content in Figure 3b–e. 5-membered rings were the most common for these compositions. For the compositions with constant SiO_2_ contents (Figure 3b,c), those with higher R=K2O(CaO+K2O) ratios tend to have more 5-membered rings but less 6-membered rings. This is most clearly seen when the binary compositions of Figure 3d. The dominance of 5-membered rings has been reported from interpretations of experimental neutron-scattering measurements of charge-compensated Al_2_O_3_–CaO–SiO_2_ glasses with similar SiO_2_ contents^15^ and is discussed further when structural properties of the rings are presented.

A preference for large rings (>12-membered) of a particular size range is also apparent for most of the compositions. These preferential ring sizes are slightly larger for the compositions with the higher R=K2O(CaO+K2O) ratio. Given that substitution of one Ca cation requires two K cations on a charge basis, and that the ionic radius of the latter is larger, these differences in ring distributions reflect the spatial distribution of SiO_4_ tetrahedra becoming more segregated with higher R=K2O(CaO+K2O) ratios; i.e., they tend to be either spatially dense or sparse. These differences reflect those reported for soda-lime glass simulations with varying cation contents^17^ and represent the sizes of the basic cation clusters that are formed in each composition. These features are more apparent with a decreasing SiO_2_ content, where the differences in the character of the basic cations are accentuated, especially in Figure 3d. For the compositions S50-R0.3 and S53-R0.6 (Figure 3e), their relatively high degree of depolymerization permits only formation of very few rings, though every ring size up to at least 40-membered could be found. The possibility of preferential rings of larger sizes in these compositions is not ruled out.

### 3.3 Ring Properties

The mean Si–O bond lengths and Si–O–Si angles comprising the different ring sizes in each composition are listed in Figure 4. The relatively short Si–O bond lengths and wide Si–O–Si angles that comprise 5-membered rings suggest the stability of such rings, which is in agreement with their relative abundance as shown. Overall, small 5- to 7-membered rings are the most stable and correspondingly the most abundant collectively.

**Figure 4:**
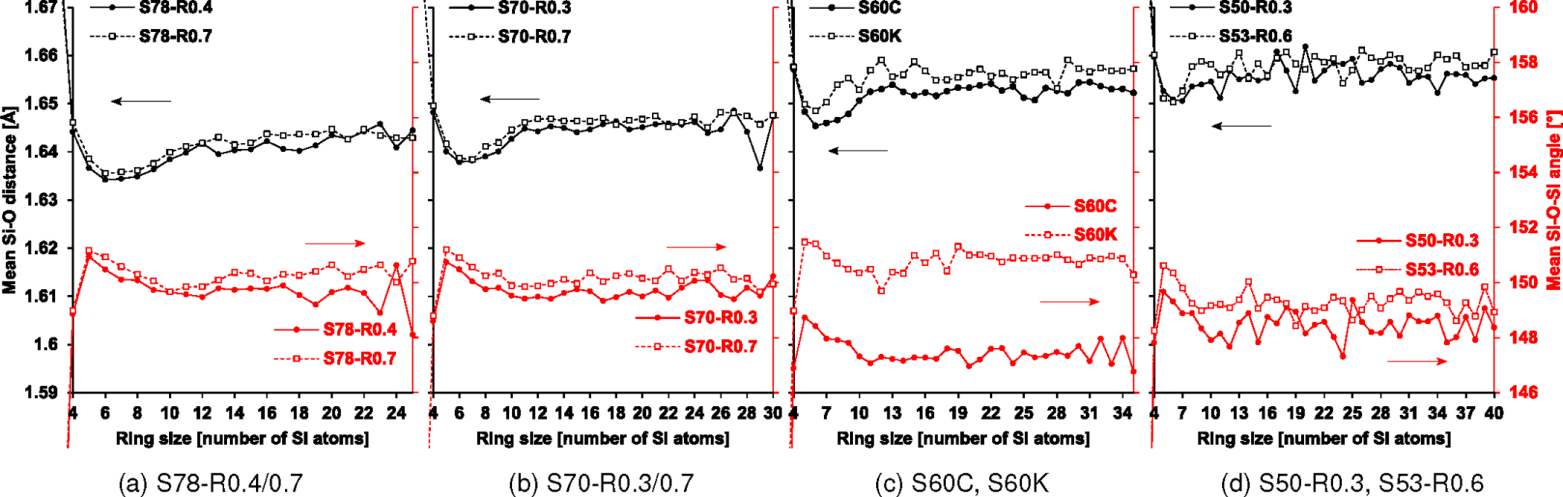
Mean Si–O bond lengths and Si–O–Si angles belonging to differently sized primitive rings for the compositions. The leftmost and rightmost *y*-axes are common to all the plots. The scarcity of 2- and 3-membered rings, which have mean Si–O bond lengths and Si–O–Si angles that are significantly longer and narrower, respectively, are omitted for clarity.

The interatomic potential used in this study is known to reproduce Si–O–Si angles that are wider than those from experimental analyses of glasses,^20^ which can lead to inaccuracies in the simulated medium-range ordering. Nonetheless, some expected trends are observed in the current results. Lodesani et al.^4^ asserted that the Si–O–Si angles should become narrower with increasing ring size as a spatial requirement to accommodate increasing numbers of SiO_4_ units. This is observed in Figure 4, where the mean Si–O–Si angles of the rings narrow from 5- to 12-membered rings. The geometrical relationship, as governed by energetic constraints between Si–O bond lengths and Si–O–Si angles, also means that their trends mirror each other to an extent.^32^ However, these structural properties do not vary systematically for rings larger than 12-membered. Note that the mean Si–O values are presented in Figure 4 rather than bond lengths based on the first peak of radial distribution functions (RDFs). The scarcity of some ring sizes meant that RDFs could not give a reliable representative value for their bond lengths. The mean value was therefore chosen for a comparison between the ring sizes. RDF evaluations based on all Si–O bonds for each simulation gave lengths of 1.60–1.63 Å, with the longer lengths found in the compositions with larger shares of CaO and K_2_O. These values are in line with reported measurements of similar CaO/K_2_O–SiO_2_ melts at high temperature.^33,34^

The mean Si–O bond lengths are slightly longer, and the mean Si–O–Si angles are slightly wider for the compositions with a higher R=K2O(CaO+K2O) ratio. These differences in the ring structure caused by Ca and K cations are most apparent in the binary compositions shown in Figure 4c. They indicate that the rings are more stretched to accommodate for the larger ionic radii of K cations. In addition, the weakening (lengthening) of Si–O bonds with increasing depolymerization is apparent. These observations are consistent with the lower viscosities as measured for these slags with higher R=K2O(CaO+K2O) ratios at constant SiO_2_ contents.^29^ A correlation between the lengthening of bridging Si–O bonds, in general, with viscosity has been shown previously,^27,35^ and is also evident for the Si–O bonds that form the rings, as demonstrated for the 4-membered to 10-membered rings in Figure 5. Qualitative logarithmic correlations can be seen between the bond lengths of the different ring sizes. It indicates that such ring structures are also weakened with depolymerization, which collectively reduces the ability of the network to resist shearing forces, leading to a lower viscosity.

**Figure 5:**
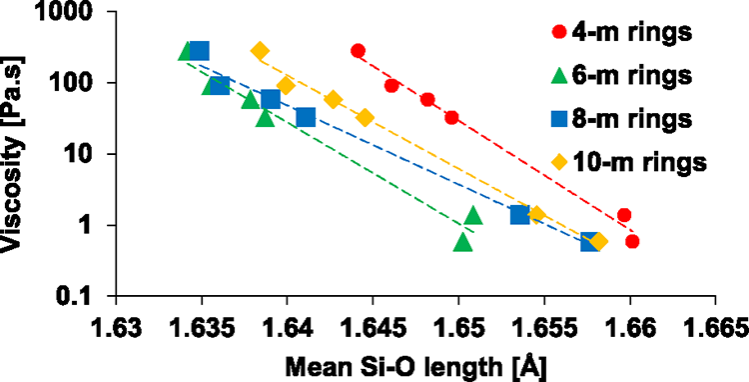
Correlation between viscosity values measured by Chen and Zhao^29^ and the Si–O bonds of different-sized rings.

The *Q*_*n*_ value denotes the number of bridging O atoms (Si–O–Si) to which a Si atom is bonded. Figure 6 shows that the mean *Q*_*n*_ value of Si atoms generally decreases with increasing ring size, which is expected because depolymerization facilitates the presence of the larger rings. Despite the differences in composition, the mean *Q*_*n*_ values of the most common small rings (5- to 8-membered) are similarly around +0.2 higher than that of the mean *Q*_*n*_ values of the largest rings. All compositions also exhibit a dip in the mean *Q*_*n*_ value of Si atoms that make up 5-membered rings. This indicates that they are generally less polymerized than those that make up the other small-sized rings. With slightly less neighboring Si atoms in competition for O atoms that can influence the ring structure, this can contribute to the stabilization of the 5-membered rings, as shown previously. Moreover, the mean *Q*_*n*_ value does not vary significantly for Si atoms in rings larger than 12-membered, which suggests that the chemical environment becomes indistinguishable for Si atoms that constitute these larger rings. The greater depolymerization effect of K_2_O compared to CaO is seen slightly in the S78 and S70 compositions and more obviously between S60C and S60K.

**Figure 6:**
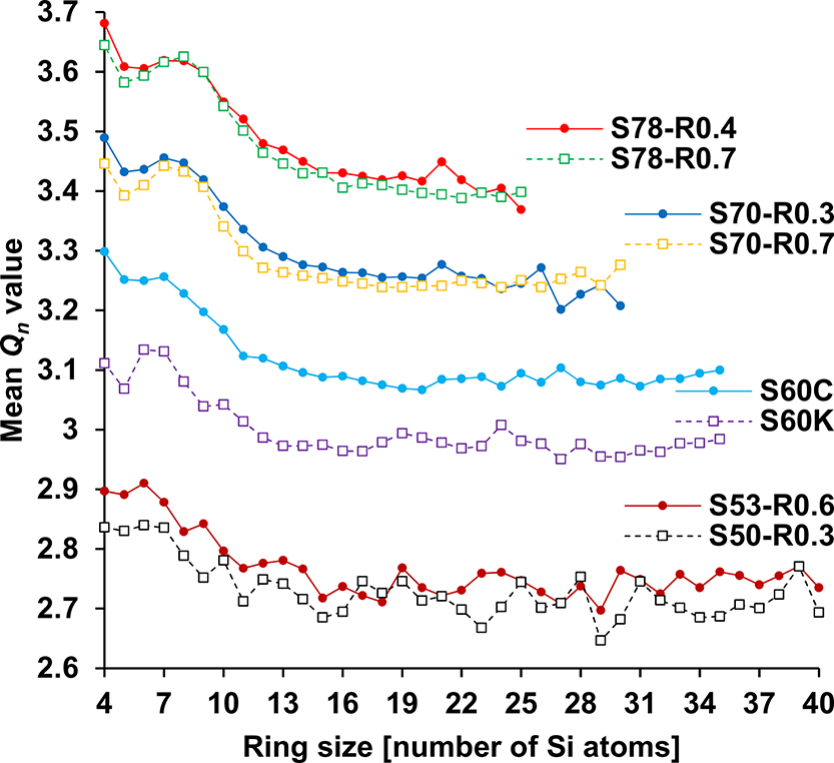
Mean *Q*_*n*_ value of Si atoms that constitute rings of different sizes in the different compositions.

### 3.4 Network-Modifier Cations inside Rings

To confirm that the variations in the primitive ring statistics are attributable to the presence of the network-modifier cations, we counted the numbers of each basic cation located inside each ring. A simple procedure was used to determine which cations were located inside a ring, as demonstrated for the ring in Figure 7a. The ring cavity is considered as the closed interior space that is traced out by each ring pathway, where occupation by the basic cations is anticipated. To define it systematically, this was considered to be the volume that a cylinder sweeps out when one end is fixed to the geometric center of the ring while rotating such that the other end passes through, in order, every Si atom that make up the ring, as shown in Figure 7b. This was intended to give a reasonable approximation of the ring shape in a systematic manner, especially in cases where the ring pathway is tortuous. Any cation with its center located within the cavity volume was considered to be located inside the ring, as exemplified in Figure 7c. Considering that the Si–O distance is approximately 1.6 Å and the size of the basic cations, a cylinder radius of 1.8 Å was deemed to give reasonable results. The sensitivity of the number of cations counted when the radius of the cylinder was varied between 1.6 and 2.0 Å is shown in Supporting Information Figure S3. Examples of rings with their inner cations, if any, are shown in Figure 8. For rings smaller than 7-membered, the basic cations were not found to occupy the cavities. The number of cations inside the rings is presented for the S70 compositions in Figure 9a,b. The basic cations can occupy the cavities of rings that are as small as 7-membered but it is only from approximately 11-membered rings or larger that they do so consistently. This corresponds with the increase in rings of these larger sizes compared to those of pure liquid SiO_2_, as seen in Figure 3a. Thereafter, the numbers of basic cations, including their sum, that occupy ring cavities increase almost linearly with increasing ring size. Such occupation varies depending on the shape of the ring, and often, the tortuosity of the ring pathway means that the basic cations are not accommodated to their full potential. Figure 9c indicates minor differences in the total number of cations in the ring cavities between the compositions. For the S70-R0.3 composition (Figure 9a), with a K:Ca molar ratio of about 1, a comparatively large increase in the standard variations of the cation numbers occurs for rings larger than 26-membered. This is due to the preferential self-clustering behavior exhibited by each respective basic cation, especially K.^11,27^ This means that these rings tend to enclose either K-rich regions or Ca-rich regions. In contrast, the abundance of K compared to Ca in the S70-R0.7 composition suppresses this occurrence, which is reflected in the smaller corresponding standard variations seen in Figure 9b. The larger rings in compositions with higher R=K2O(CaO+K2O) are consistent with wide percolation channels that facilitate the more rapid diffusion of K cations. The numbers of cations in the ring cavities from all the simulations are found in Supporting Information Figure S2.

**Figure 7:**
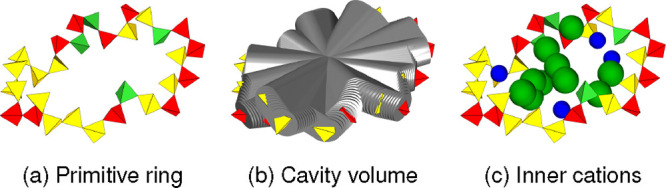
Cavity volume of the example ring in panel (a) is assumed to be equivalent to the combined volume depicted by the gray cylinders in panel (b). Any cations with centers inside this volume are considered to be located inside the ring, as in panel (c).

**Figure 8:**
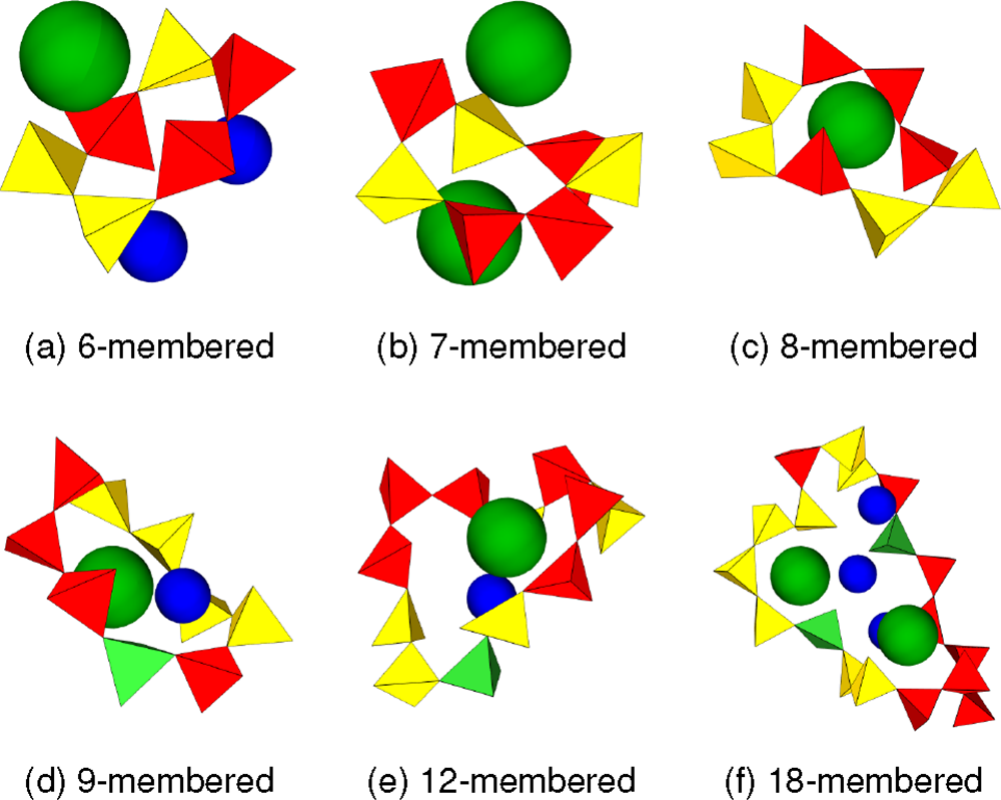
Examples of primitive rings with inner Ca (blue) and/or K (green) cations. The 6- and 7-membered rings contain no cations inside but the nearest cations are shown for comparison. The cation radii are based on values from Whittaker and Muntus.^36^

**Figure 9:**
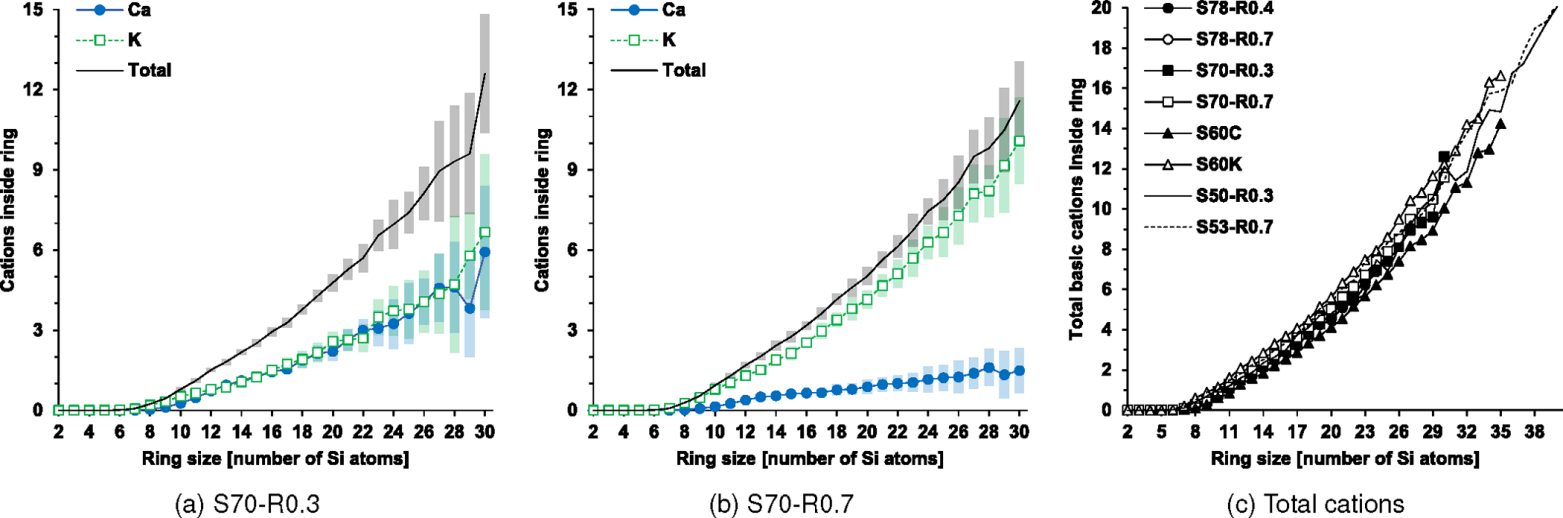
(a,b) Number of basic cations in silicate rings in the compositions with 70 mol % SiO_2_. Shaded bars represent standard deviations. (c) Total number of basic cations in all compositions.

It is worth noting that these results confirm that primitive rings are effective in finding regions of network-modifier cation clusters in silicate systems. Primitive rings that can be decomposed into more than two smaller rings but without creating shortcuts (i.e., rings that enclose a net-like structure^13^) were rare or nonexistent; otherwise, a pervasive absence of basic cations would create larger standard deviations in their totals.

### 3.5 Interconnectivity between Rings

To supplement the ring size distributions presented, a topological representation of the spatial distribution of the rings in relation to each other is needed. Le Roux and Jund^37^ presented a novel method that distinguishes the essential topology of rings that are contained within a network. Here, we use a similar but more intuitive approach that is based on the definition of a local cluster at each SiO_4_ unit by Marians and Hobbs.^30^ Accordingly, the sizes and numbers of rings that belong to a particular SiO_4_ unit (i.e., the local cluster) are counted. For example, consider the gray SiO_4_ unit that hosts a local cluster connecting 1 × 7-membered, 2 × 6-membered, and 3 × 5-membered rings, as shown in Figure 10.

**Figure 10:**
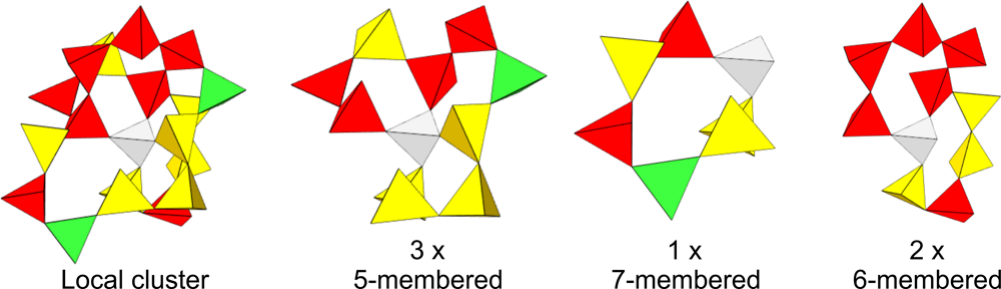
Interconnectivity of different-sized primitive rings intersecting at the gray SiO_4_ tetrahedron, which is quantified by the interconnectivity data in Table 2. The left shows the entire interconnected local cluster, while the others show decompositions based on each ring size.

From the perspective of each of the three five-membered rings, they are each interconnected with 2 × 5-membered, 2 × 6-membered, and 1 × 7-membered rings via the gray SiO_4_ unit. Likewise, for each of the two 6-membered rings: they are each interconnected to 3 × 5-membered, 1 × 6-membered, and 1 × 7-membered rings. For the 7-membered ring, it is interconnected to 3 × 5-membered and 2 × 6-membered rings. This information can be tabulated for each Si atom, as presented in Table 2. It describes the quantities that each ring size is interconnected to, with respect to each ring size. The interconnectivity table of every SiO_4_ unit in the simulation domain can be summed together within the table and, when divided by the total number of Si atoms, it gives the average interconnectivity between each ring size that can be compared between the different compositions. In other words, it indicates the interconnectivity tendencies between different ring sizes, if any, within the silicate network topology.

**Table 2:** Interconnectivity Table for the Gray SiO_4_ Tetrahedron in Figure 10, Which Interconnects 3 × 5-Membered, 1 × 7-Membered, and 2 × 6-Membered Rings

	interconnecting ring size							
ring size	2	3	4	5	6	7	8	⋯
	number of interconnecting rings							
2	0	0	0	0	0	0	0	⋯
3	0	0	0	0	0	0	0	⋯
4	0	0	0	0	0	0	0	⋯
5	0	0	0	2	2	1	0	⋯
6	0	0	0	3	1	1	0	⋯
7	0	0	0	3	2	0	0	⋯
8	0	0	0	0	0	0	0	⋯
⋮	⋮	⋮	⋮	⋮	⋮	⋮	⋮	⋱

The interconnectivity table has several features. First, by directly and only counting the number of rings that are interconnected, values are proportional to their degree of interconnectivity. This should allow for intuitive interpretation of the table, especially when visualized in graphical form, e.g., a heatmap. Second, the table is a nonsymmetrical matrix in general, which means that it can convey subtle differences in the interconnectivity. For the example in Table 2, it can be interpreted that 7-membered rings are likely to be interconnected with multiple 5- and 6-membered rings, but the opposite is not equal: the presence of the latter two are associated with a lesser degree with 7-membered rings. Finally, SiO_4_ units that belong to only one ring are not interconnected by definition and are not counted in the interconnectivity table. The interconnectivity table can also be applied to other ring definitions and compositions that involve network-forming elements besides Si, possibly serving as a fingerprint to differentiate between networked structures. The features and interpretation of the interconnectivity table are demonstrated by applying it to the simulation results.

The interconnectivity of primitive rings in the S100 melt is shown in Figure 11a. The ring sizes with the highest interconnectivity are 5- to 8-membered, which are on average interconnected with up to 3.5 rings that are mostly within this same size range. The general symmetry of the off-diagonal values across the diagonal line (bottom left to top right) reflects the general homogeneity of the topology. In other words, the tendency for interconnectivity between the rings is reciprocated.

**Figure 11:**
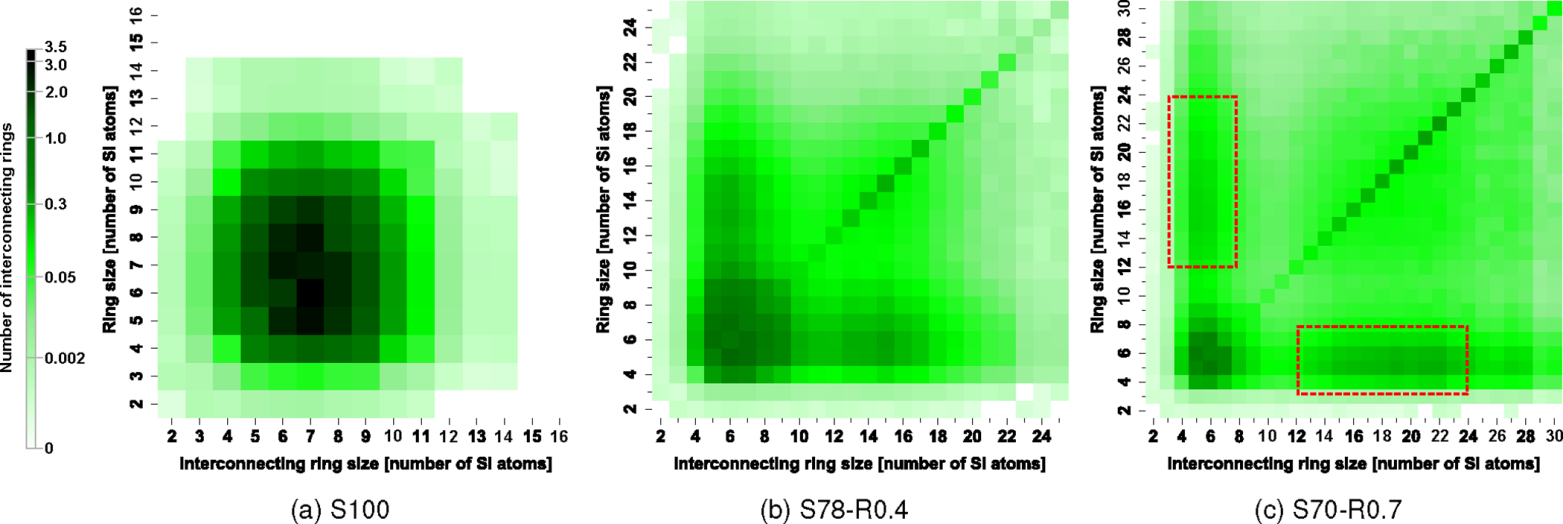
Heatmaps depicting the interconnectivity of ring sizes in selected simulations. Note the nonlinearity of the color indicator to enhance comparison. The asymmetry in interconnectivity is most apparent in panel (c) as marked by the dashed red boxes.

The effect of Ca and K cations on the interconnectivity is illustrated by the compositions in Figure 11b,c. The lower intensities of these heatmaps, overall, reflect the decreased polymerization of these compositions compared to pure SiO_2_. Even so, the interconnections between the smaller rings are the most abundant. Compared to the S100 composition, the range of the most interconnected ring sizes decreases to 5- to 7-membered rings for the S78-R0.4 composition, while this decreases to 5- and 6-membered rings for the S70-R0.7 composition. Rings of these sizes generally do not contain any basic cations inside them. Hence, these ring clusters represent Si-rich regions within the silicate network.

Figure 11b,c also indicates that these ring sizes also exhibit a tendency to be interconnected to larger rings of a specific size range. These larger ring sizes are centered at around 15- and 20-membered for the S78-R0.4 and S70-R0.7 compositions, respectively, which correspond to the minor peaks seen in the ring size distributions of Figure 3. Topologically, this means that basic cation clusters of particular sizes, as dictated by the composition, tend to, on average, occur directly adjacent to regions that are Si-rich. This feature is slightly enhanced in compositions containing more K cations, and is likely due to the greater tendency of K to form clusters.^27^ The size range of the larger interconnecting rings also increases with the K content. A general asymmetry of the interconnectivity heatmap across the diagonal is present, most evident in Figure 11c. This is due to a higher occurrence of the smaller rings being interconnected to the larger rings rather than vice versa (roughly 2× in this case). Topologically, this suggests that the smaller rings are located intermittently around the larger rings. This also implies that the Si-rich regions, where these smaller rings reside, have a tendency to be located adjacent to alkali clusters of sizes that are dependent on the composition. For the S70-R0.7 composition, Figure 9b shows that this corresponds to approximately five basic cations. This asymmetric topological feature is slightly more apparent in compositions with more K cations.

The heightened interconnectivity between these smaller rings and these larger rings means that members of the former can act as alternate pathways for the latter. An example of this was illustrated by gray SiO_4_ tetrahedron in Figure 2, which acts as a hub that interconnects eight 15-membered rings via pathways of smaller-sized ring. These large rings with alternate pathways are counted as separate rings, increasing their abundance and the numbers of Si atoms that constitute them. Because of this, the same-size interconnectivity is enhanced for these large rings, as indicated by the prominent intensities along the diagonals of the heatmaps. In contrast, there is a lack of prominence in the diagonal values in the case of S100 due to the prevalence of relatively small rings with tendencies to be highly interconnected with each other. This dominates over the aforementioned interconnectivity biases associated with the larger rings that are present in the other compositions. It is also noted for the compositions containing basic cations that while interconnectivity between small-sized (4- to 7-membered) rings is common, larger rings generally do not exhibit such interconnectivity with rings that are similar in abundance but slightly different in size, as indicated by the low intensities directly adjacent to the diagonal. The interconnectivity plots of all the compositions are compared in Supporting Information Figure S4, and their representation with an alternate color palette can be found in Figure S5.

The composition of biomass-based slags can vary significantly and involve many other elements, including similar network-modifying elements such as Na and Mg, as well as amphoteric elements such as Al and Ti. These elements behave and affect the silicate network differently depending on the composition,^38,39^ making it of interest to conduct similar studies to elucidate their impacts on the clustering of cations and connectivity of the network.

## 4 Conclusions

The statistics and structures of primitive rings in silicate melts containing K and Ca have been studied. The primitive ring definition was shown to be effective in finding regions where clusters of these network-modifying cations can occupy, as supported by visualizations and by the quantification of cations located inside ring cavities. The ring distributions for compositions with higher R=K2O(CaO+K2O) ratios contain larger rings, likely due to a combination of the larger ionic radius of K and its higher numerical abundance on a charge basis. Compositions with more K cations also produce rings with longer Si–O bonds and wider Si–O–Si angles; i.e., rings that are more stretched. Small rings were shown to have the strongest interconnectivity with each other while also possessing a tendency to be interconnected to larger rings of specific sizes, depending on the composition.

## Supplementary Material



## Notes

Primitive ring statistics without compensation for ring size (Figure S1); numbers of cations counted in the rings of the relevant cases (Figure S2); sensitivity analysis of the cylinder radius used in counting ring cations (Figure S3); heatmaps depicting the interconnectivity between ring sizes from all cases (Figure S4); and heatmaps depicting the interconnectivity between ring sizes from all cases (alternate color palette) (Figure S5) (PDF)
