# Supplementary material for: Silicate Rings in Woody Biomass Ash Melts Based on Molecular Dynamics Simulations
Source: ACS Omega. 2026 Mar 8;11(11):17907–15. doi: 10.1021/acsomega.5c12582 (PMC13019399; doi:10.1021/acsomega.5c12582)
Supplement: Supplementary file 1 [file ao5c12582_si_001.pdf]

Silicate rings in woody biomass ash melts based on molecular dynamics simulations

Charlie Ma[a,*]

[a] *Thermochemical Energy Conversion Laboratory (TEC-Lab), Department of Applied Physics and Electronics, Umeå University, Umeå, SE-901 87, Västerbotten, Sweden*

This supporting information file contains the following:

- S1: Primitive ring statistics without compensation for ring size
- S2: Numbers of cations counted inside rings from the relevant cases
- S3: Sensitivity analysis of the cylinder radius used in counting ring cations
- S4: Heatmaps depicting the interconnectivity between ring sizes from all cases
- S5: Heatmaps depicting the interconnectivity between ring sizes from all cases (alternate colour palette)

Other information can be made available upon request. Contact: charlie.ma@umu.se

*Corresponding author
Email address: charlie.ma@umu.se (Charlie Ma)

1. Supporting information

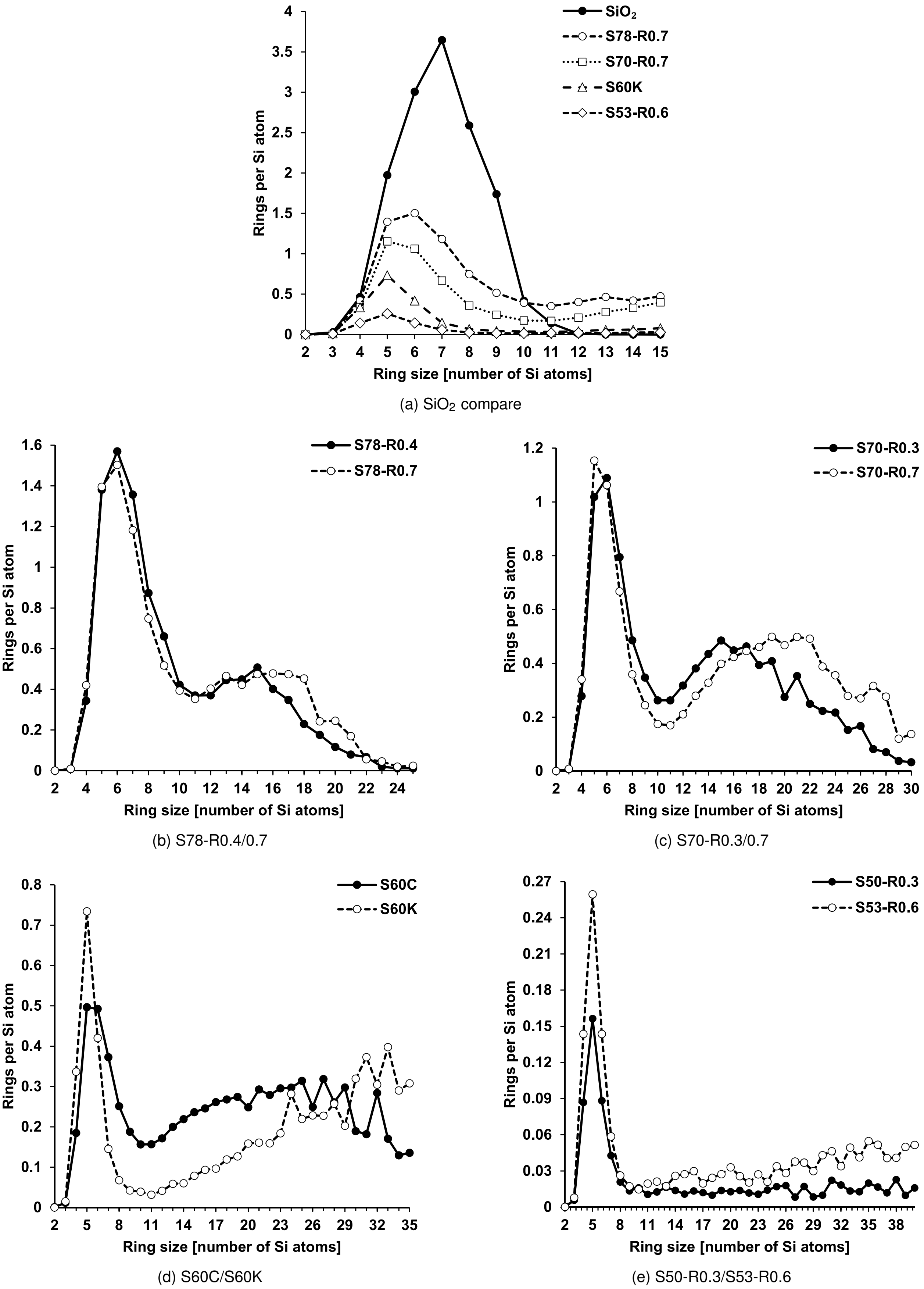

(a) SiO$_2$ compare

(b) S78-R0.4/0.7

(c) S70-R0.3/0.7

(d) S60C/S60K

(e) S50-R0.3/S53-R0.6

Figure S1: Primitive ring statistics without adjusting for ring size, as per Yuan and Cormack [1]

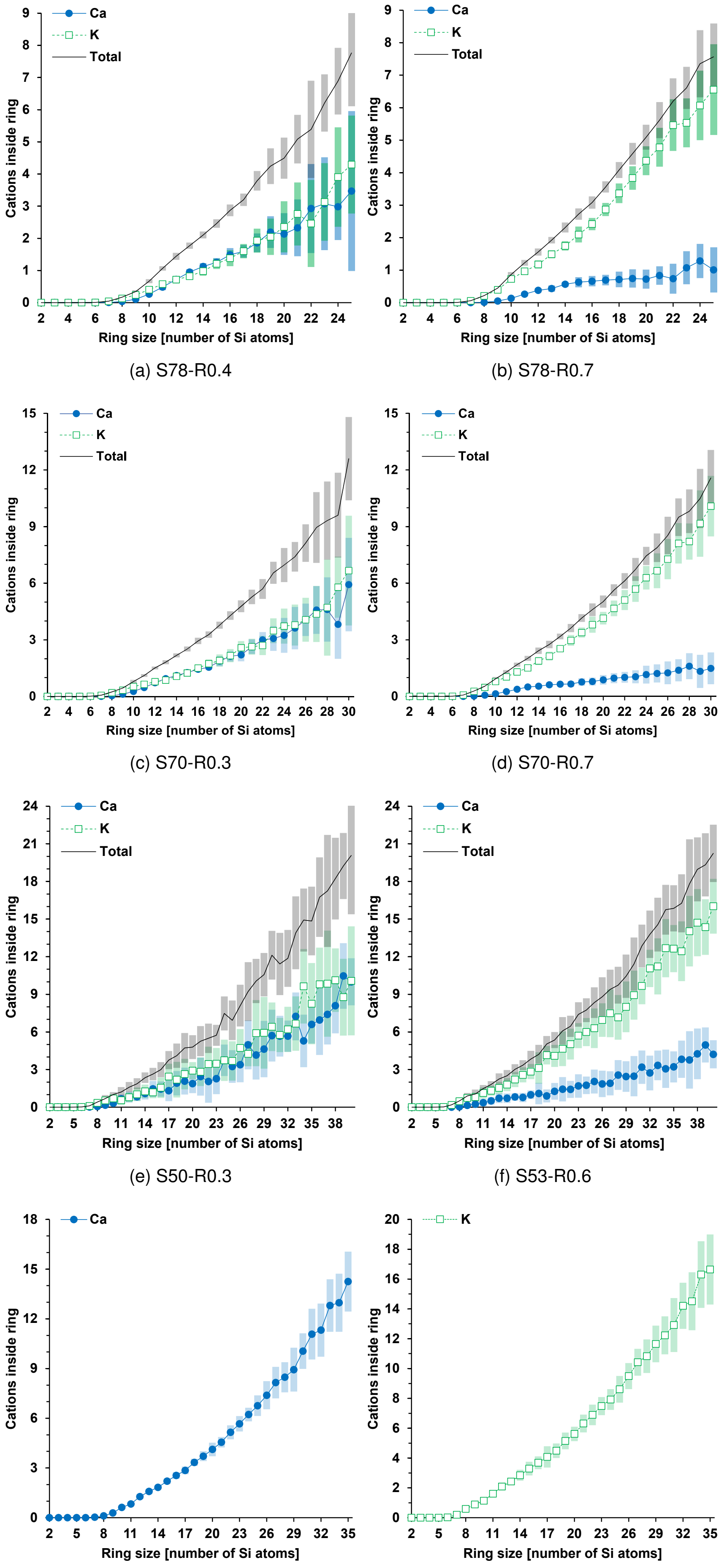

(a) S78-R0.4 (b) S78-R0.7

(c) S70-R0.3 (d) S70-R0.7

(e) S50-R0.3 (f) S53-R0.6

(g) S60C (h) S60K

Figure S2: The number of basic cations occupying the cavities of primitive rings from each simulation, based on the method depicted in Fig. 7. Note the different axes belonging to each plot. For the compositions with lower R = $K_2O/(CaO + K_2O)$ ratios where the numbers of Ca and K cations are more similar, the variations in the numbers of each cation found in ring cavities are generally larger, reflecting the tendency of preferential clustering behaviour pertaining to each cation.

Figure S2

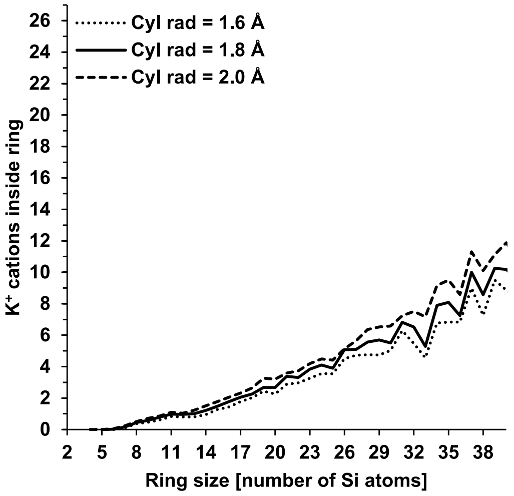

(a) Number of K cations

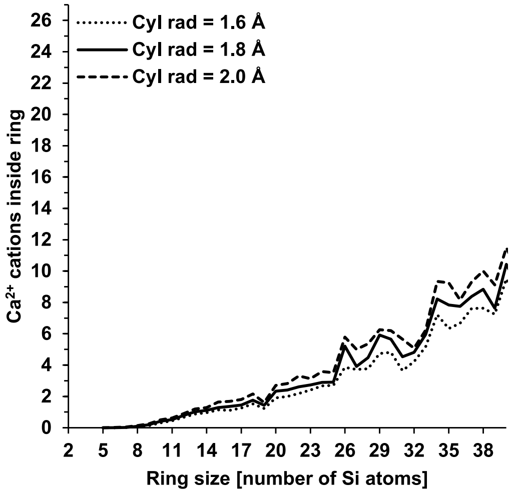

(b) Number of Ca cations

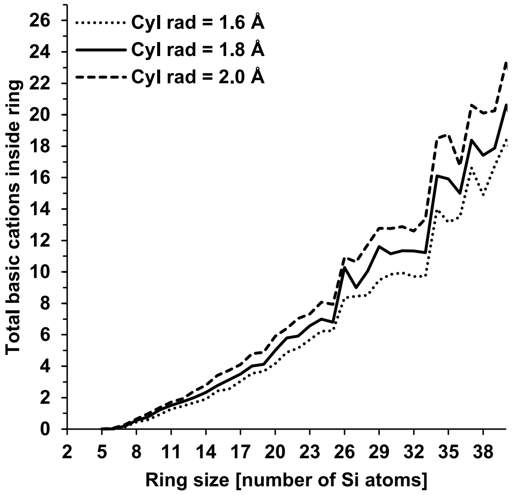

(c) Total number of cations

Figure S3: The influence of the cylinder radius used in the method described in Sec. 3.4 to count the number of basic cations within a ring cavity.

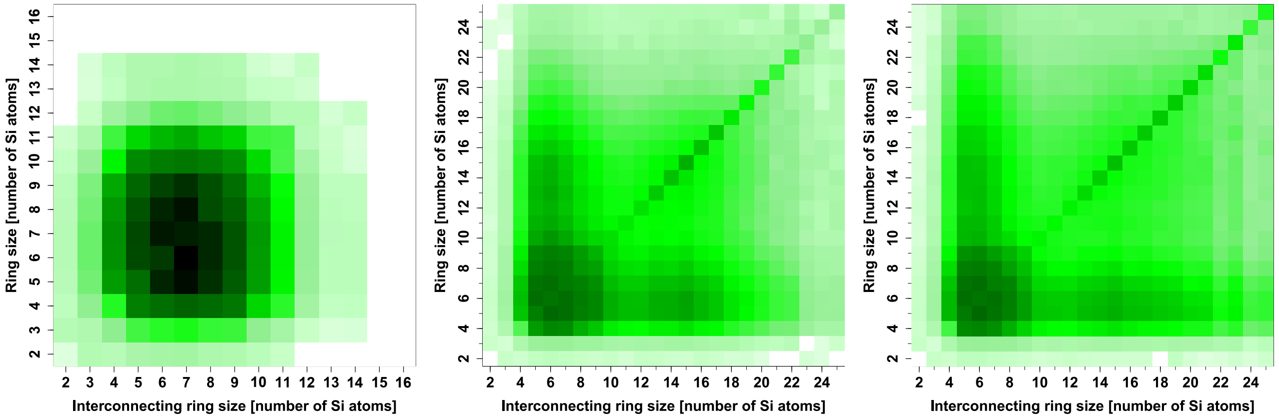

(a) S100 (b) S78-R0.4 (c) S78-R0.7

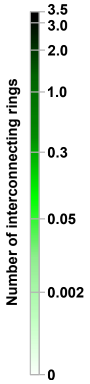

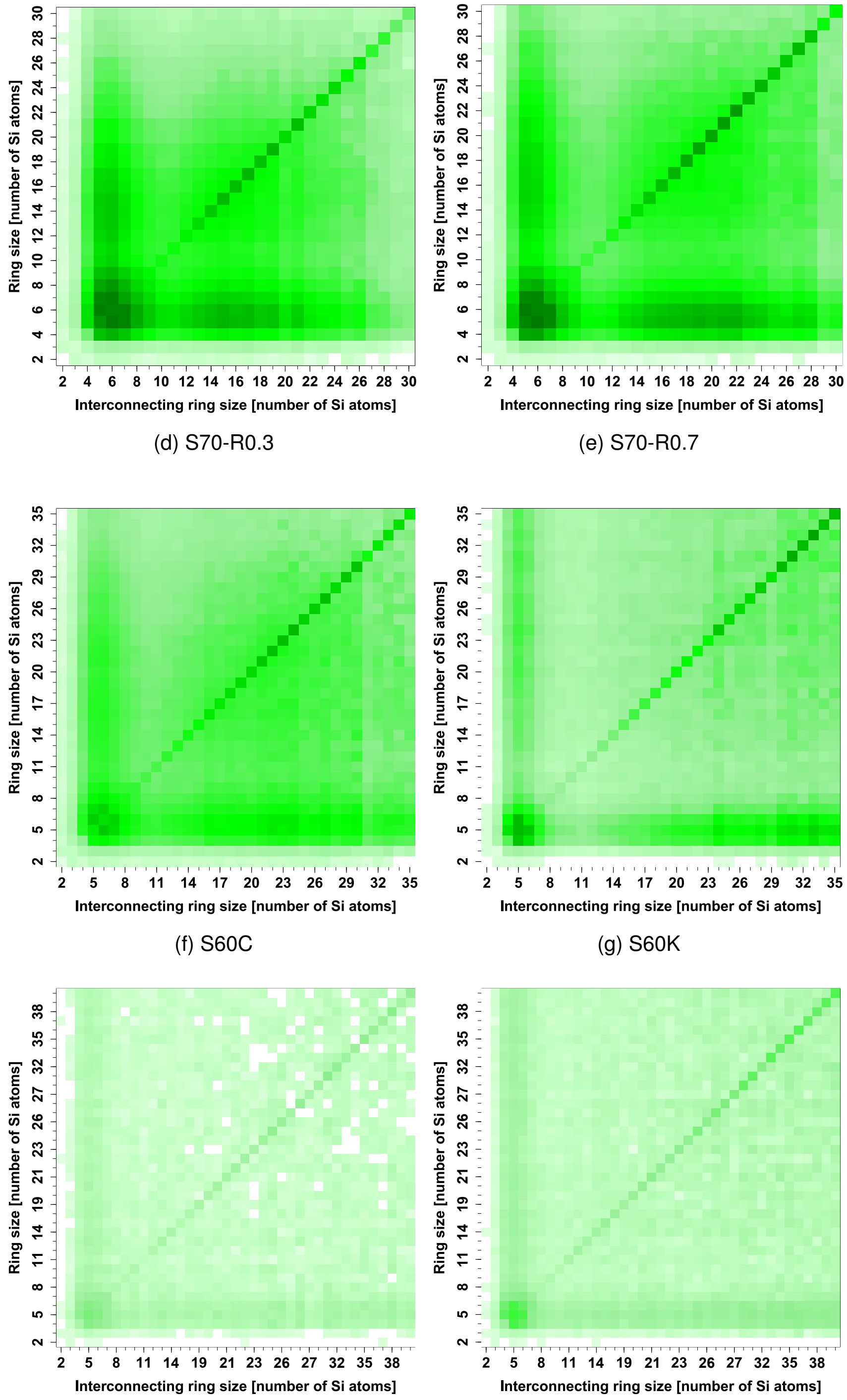

(d) S70-R0.3 (e) S70-R0.7

(f) S60C (g) S60K

(h) S50-R0.3 (i) S53-R0.6

Figure S4: Heatmaps depicting the interconnectivity between ring sizes in each simulation. Their overall intensities reflect the degree of polymerisation in each composition. 5- to 7-membered rings in the compositions containing basic cations, (b) to (i), have a tendency to be interconnected to larger rings of a specific size range, the latter of which increases with depolymerisation. This means that basic cation clusters of a certain size, as dictated by the composition, tend to occur adjacent to regions that are Si-rich. This also indicates the possibility of numerous alternate pathways for these larger rings, which manifest in intensities along the diagonals of the heatmaps because the constituting members are all same-size interconnected. There is a general asymmetry of the heatmaps across this diagonal due to a higher occurrence of small rings being interconnected to larger rings rather than vice versa. It suggests that the smaller rings are located intermittently around the larger rings. These topological features are more evident in compositions containing more K cations.

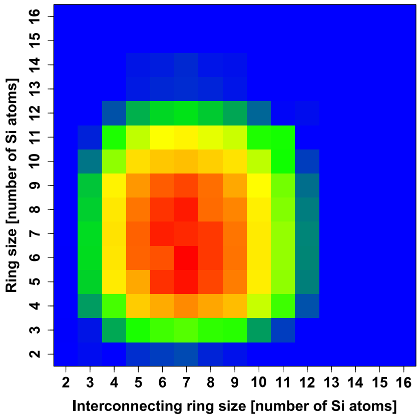

(a) S100

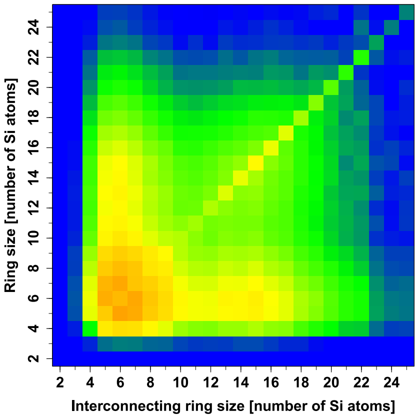

(b) S78-R0.4

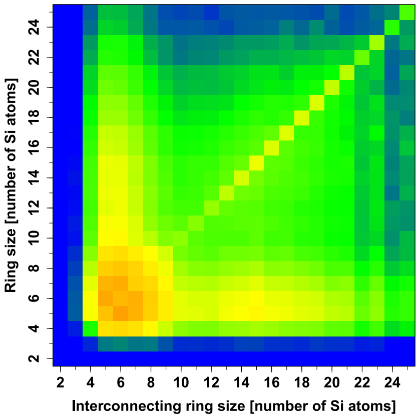

(c) S78-R0.7

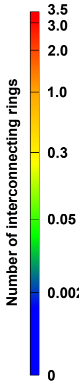

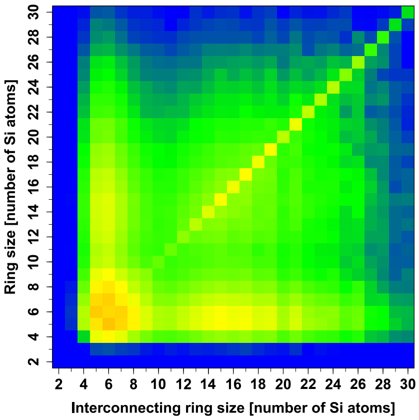

(d) S70-R0.3

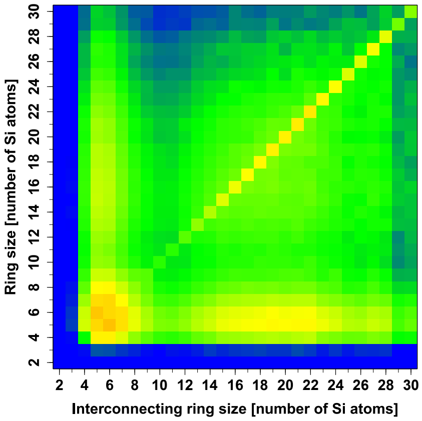

(e) S70-R0.7

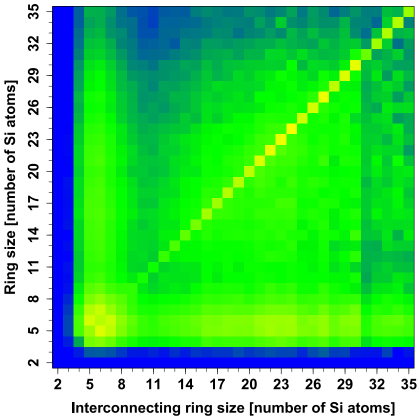

(f) S60C

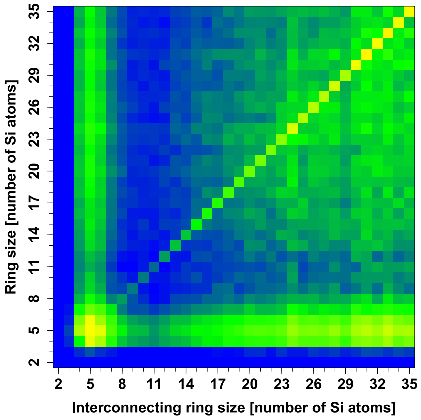

(g) S60K

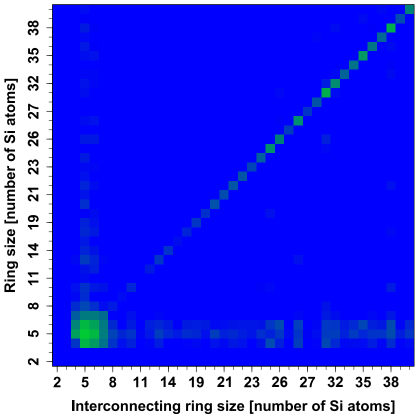

(h) S50-R0.3

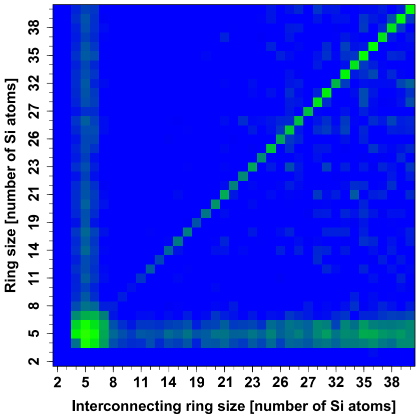

(i) S53-R0.6

Figure S5: Heatmaps based on an alternate colour palette depicting the interconnectivity between ring sizes in each simulation as per Figure S4.

Figure S5

References

[1] X. Yuan, A. Cormack, Efficient algorithm for primitive ring statistics in topological networks, Computational Materials Science 24 (2002) 343–360.
